# Tannic Acid-Mediated Aggregate Stabilization of Poly(*N*-vinylpyrrolidone)-*b*-poly(oligo (ethylene glycol) methyl ether methacrylate) Double Hydrophilic Block Copolymers

**DOI:** 10.3390/nano9050662

**Published:** 2019-04-26

**Authors:** Noah Al Nakeeb, Ivo Nischang, Bernhard V.K.J. Schmidt

**Affiliations:** 1 Max-Planck Institute of Colloids and Interfaces, Department of Colloid Chemistry, Am Mühlenberg 1, 14476 Potsdam, Germany; noah.alnakeeb@gmail.com; 2 Laboratory of Organic and Macromolecular Chemistry (IOMC), Friedrich Schiller University Jena, Humboldtstraße 10, 07743 Jena, Germany; 3 Jena Center for Soft Matter (JCSM), Friedrich Schiller University Jena, Philosophenweg 7, 07743 Jena, Germany

**Keywords:** block copolymer self-assembly, analytical ultracentrifugation, tannic acid

## Abstract

The self-assembly of block copolymers in aqueous solution is an important field in modern polymer science that has been extended to double hydrophilic block copolymers (DHBC) in recent years. In here, a significant improvement of the self-assembly process of DHBC in aqueous solution by utilizing a linear-brush macromolecular architecture is presented. The improved self-assembly behavior of poly(*N*-vinylpyrrolidone)-*b*-poly(oligo(ethylene glycol) methyl ether methacrylate) (PVP-*b*-P(OEGMA)) and its concentration dependency is investigated via dynamic light scattering (DLS) (apparent hydrodynamic radii ≈ 100–120 nm). Moreover, the DHBC assemblies can be non-covalently crosslinked with tannic acid via hydrogen bonding, which leads to the formation of small aggregates as well (apparent hydrodynamic radius ≈ 15 nm). Non-covalent crosslinking improves the self-assembly and stabilizes the aggregates upon dilution, reducing the concentration dependency of aggregate self-assembly. Additionally, the non-covalent aggregates can be disassembled in basic media. The presence of aggregates was studied via cryogenic scanning electron microscopy (cryo-SEM) and DLS before and after non-covalent crosslinking. Furthermore, analytical ultracentrifugation of the formed aggregate structures was performed, clearly showing the existence of polymer assemblies, particularly after non-covalent crosslinking. In summary, we report on the completely hydrophilic self-assembled structures in solution formed from fully biocompatible building entities in water.

## 1. Introduction

Block copolymer self-assemblies play a prominent role in current polymer science [1,2]. Self-assemblies are applied in many fields of research such as (nano)-lithography [3,4], nanoparticle formation [5,6], and catalysis [7,8], but also in the biomedical field, where applications such as imaging [9,10], biological sensing [11,12], and drug delivery [13,14,15] are investigated. Amphiphilic block copolymers are utilized frequently for these tasks in an aqueous environment, e.g., via self-assembly to micelles, vesicles, or more complex structures [16,17]. Recently, the formation of amphiphilic self-assembled structures was shifted to the polymerization process [18,19]. In such a way, the aforementioned and more complex structures can be accessed in one step via the adjustment of monomer conversion. Notwithstanding, self-assemblies of amphiphilic block copolymers in aqueous solution face some disadvantages, e.g., low permeability [20]. Particularly, in the prospected applications of nanoreactors, permeability is a key property to enable efficient reaction progress. One solution to this problem is the introduction of artificial protein channels [21,22]. Another option is the implementation of stimuli-responsive blocks that allow selective swelling/deswelling of the hydrophobic domain [23,24]. 

Another way would be to switch from an amphiphilic block copolymer to a completely water-soluble block copolymer. Frequently, one of the utilized blocks is stimuli-responsive, which facilitates self-assembly. As the solubility changes after stimulus application in most cases, the self-assembled structures are not purely hydrophilic anymore. In the case of self-assembly based on the hydrophilic effect, no stimulus is employed to form self-assembled structures via a change in solubility. The self-assembly process of double hydrophilic block copolymers (DHBCs) in water can be related to the macroscopic two-phase formation of hydrophilic homopolymer mixtures [25], e.g., poly(ethylene glycol) (PEG) and dextran—two water soluble polymers that were utilized to purify proteins [26]—or in the formation of water-in-water emulsions [27,28]. As the blocks are connected covalently in the DHBC, no macroscopic demixing is possible; rather, a microphase separation occurs, which is termed the hydrophilic effect [29]. The process of demixing, and consequently weak aggregation, is related to the difference in hydrophilicity that leads to non-symmetric solvation. Therefore, a difference in osmotic pressure is present between the respective polymer domains, which has to be compensated via the aggregation of the polymer domains. Certainly, the architecture of the individual blocks has a significant effect as well due to interactions between the blocks and their structure in space, which can also be related to varying solvation. Recently, several examples of DHBC self-assembly were introduced, e.g., pullulan-*b*-poly(*N*,*N*-dimethylacrylamide) [30], poly(2-ethyl-2-oxazoline)-*b*-poly(*N*-vinylpyrrolidone) (PEtOx-*b*-PVP) [31], PEG-*b*-glycopolymer [32], poly(oligo(ethylene glycol) methyl ether methacrylate)-*b*-glycopolymer (POEGMA-*b*-glycopolymer) [33,34] or PEtOx-*b*-PEG [35]. Moreover, the effect of DHBC architecture was investigated, and it could be shown that a linear-brush DHBC showed significantly enhanced self-assembly behavior, i.e., the system pullulan-*b*-P(OEGMA) [36]. In addition, DHBCs were utilized in the formation of inorganic and metal–organic mesocrystals [37,38]. Considering the possible future application of DHBC in the biomedical field, a focus is on the utilization of biocompatible polymers, namely PEG and (PVP as a possible combination of particular interest [20,39]. For example, PVP is frequently used in drug formulations [40]. However, the self-assembly of linear PEG-*b*-PVP showed rather limited success [41]. Therefore, exchanging PEG with P(OEGMA) is a useful alternative, as recently shown by our group [42]. Nevertheless, one of the major challenges for DHBC self-assembly is the highly dynamic and thus poor stability of aggregates, especially under conditions of dilution. The stability of DHBC aggregates can be expressed with an equilibrium-like state between assembled aggregates and unimers in aqueous solution. The equilibrium strongly depends on concentration, and thus disassembly is observed in diluted solution. An option to circumvent the dynamics of DHBC self-assembly is the crosslinking of the formed structures at higher concentration, which renders the aggregates stable under dilution [41]. So far, covalent crosslinking was the focus of research, which essentially freezes the dynamics of the DHBC self-assembly system without the option of disassembly. A triggered disassembly could be achieved via dynamic covalent chemistry based on disulfide or imine bonds [43]. To obtain a true adaptive system, non-covalent crosslinking chemistries have to be introduced, i.e., supramolecular bonding, for example via hydrogen bonds. 

The concept of supramolecular chemistry allows the introduction of dynamics into molecular systems [44,45], e.g., via hydrogen bonding [46], host–guest complexes [47], or metal complexation [48]. Supramolecular interactions are particularly interesting for reversible crosslinking [49,50]. In that regard, tannic acid (TA) is a very useful compound to non-covalently crosslink the formed self-assemblies from PVP-*b*-P(OEGMA), as the interaction of tannic acid and PVP is well known not only from science, but also from the fining of red wine. It readily forms hydrogen bonds due to its acidic phenolic hydroxyls with a significant number of molecules, such as proteins and polymers, including PVP [51], which can be exploited in the formation of nanostructures [52,53,54]. Moreover, TA belongs to the group of tannins and can be found in wine, beer, tea, and nuts. Therefore, TA represents a natural and renewable product that is also approved by the Food and Drug Administration. Therefore, TA gained a lot of attention recently, last but not least because of its utilization in biomedical applications [55,56].

**Scheme 1:**
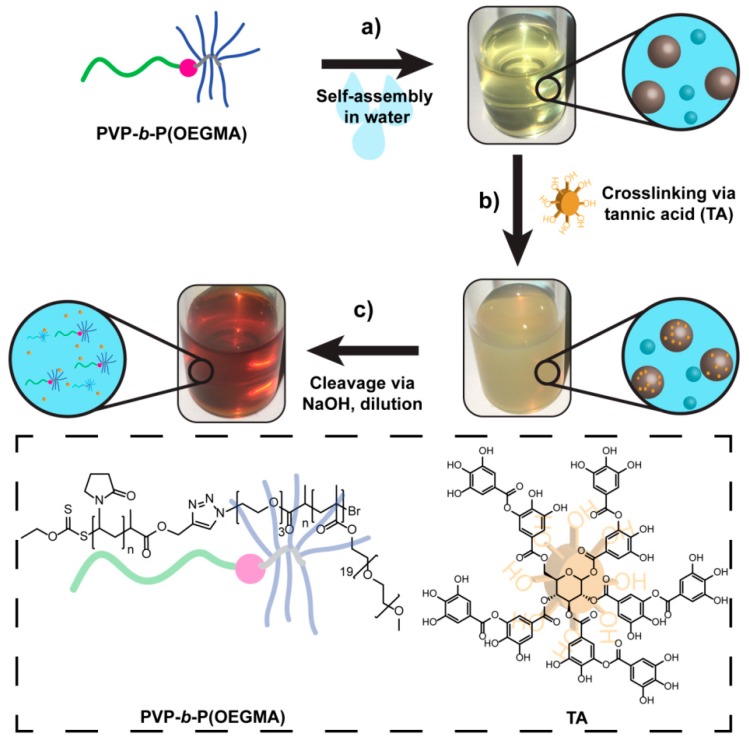
Overview of the concept utilized in the present work. (**a**) The self-assembly process of the linear-brush double hydrophilic block copolymers (DHBC) poly(*N*-vinylpyrrolidone)-*b*-poly(oligo(ethylene glycol) methyl ether methacrylate) (PVP-*b*-P(OEGMA)) in water, followed by (**b**) crosslinking of the poly(*N*-vinylpyrrolidone) (PVP) blocks via tannic acid (TA), and finally (**c**) the disassembly process via the addition of NaOH. Inset: Structures of PVP-*b*-P(OEGMA) and TA (idealized).

Herein, the self-assembly of PVP-*b*-P(OEGMA) and crosslinking via biocompatible TA in aqueous solution is described (Scheme 1). The DHBC is synthesized via a combination of reversible deactivation radical polymerization and copper (I) catalyzed azide alkyne cycloaddition (CuAAc). Subsequently, dynamic light scattering (DLS), cryogenic scanning electron microscopy (cryo-SEM), and analytical ultracentrifugation (AUC) are utilized to investigate the desired aggregate formation. The selection of these techniques was based on the different insights provided based on the physical principle of their operation. Therefore, self-assembly efficiency and aggregate sizes are studied as well as the dynamics of the formed aggregates before and after non-covalent crosslinking.

## 2. Materials and Methods 

Methods: ^1^H- and ^13^C-NMR spectra were recorded at ambient temperature at 400 MHz for ^1^H and 100 MHz for ^13^C with a Bruker Ascend400 (Bruker, Billerica, MA, USA). Dynamic light scattering (DLS) was performed using an ALV-7004 Multiple Tau Digital Correlator (ALV, Langen, Germany) in combination with a CGS-3 Compact Goniometer (ALV, Langen, Germany) and a HeNe laser (Polytec, 34 mW, *λ* = 633 nm at *θ* = 90° setup for DLS). Sample temperatures were adjusted to 25 °C and toluene was used as the immersion liquid. Apparent hydrodynamic radii (*R*_app_) were determined by LV-Correlator Software Version 3.0. Autocorrelation functions measured at a scattering angle of 90° were analyzed using Constrained Regularization Method for Inverting Data (CONTIN). Cryogenic scanning electronic microscopy (cryo-SEM) was performed on a Jeol JSM 7500 F (Tokio, Japan) and the Alto 2500 cryo-chamber from Gatan (Munich, Germany). Size exclusion chromatography (SEC) for PVP, P(OEGMA), and PVP-*b*-P(OEGMA) were conducted in *N*-methyl pyrrolidone (NMP) (99%, Carl Roth) with 0.05 mol L^−1^ of LiBr and methyl benzoate as internal standard at 70 °C using a column system with a GRAM 100/1000 column (8 × 300 mm, 7-μ particle size) from PSS (Mainz, Germany), a GRAM precolumn (8 × 50 mm) from PSS, a Shodex RI-71 detector, and a poly(methyl methacrylate) (PMMA) calibration with standards from PSS. Fourier transform infrared (FT-IR) spectra were acquired on a Nicolet iS 5 FT-IR spectrometer (Thermo Fisher Scientific, Schwerte, Germany).

Sedimentation velocity experiments were performed with a ProteomeLab XL-I analytical ultracentrifuge (Beckman Coulter Instruments, Brea, CA, USA), using double-sector epon centerpieces with a 12-mm optical path length. The cells were placed in an An-50 Ti eight-hole rotor. A rotor speed of 42,000 rpm was used. The cells were filled with 420 μL of sample solution and with 440 μL of water as the reference. The experiments were conducted for 24 h at a temperature of *T* = 20 °C. Sedimentation profile scans were recorded with the interference optics (refractive index (RI)) detection system with respect to time at 2-min intervals. A suitable selection of scans was used for data evaluation with Sedfit using the ls-g*(s) model [57], i.e. by least squares boundary modeling with the implemented Tikhonov-Phillips regularization procedure and by assuming non-diffusing species. This model results in an apparent differential distribution of sedimentation coefficients, *s*. 

Materials: Ammonium chloride (99%, Carl Roth, Karlsruhe, Germany), ascorbic acid (98%, Alfa Aesar, Karlsruhe, Germany), 2-bromopropionyl bromide (97%, Sigma Aldrich, Steinheim, Germany), *t*-butyl hydroperoxide (70% solution in water, Acros Organics, Geel, Belgium), chloromethyl polystyrene resin (2.4 mmol g^−1^, TCI, Eschborn, Germany), copper (I) bromide (CuBr, 99.99%, Sigma Aldrich), copper (II) sulfate (CuSO4, 99%, Carl Roth), dichloromethane (DCM, analytical grade, Acros Organics), diethyl ether (ACS reagent, Sigma Aldrich), *N*,*N*-dimethylformamide (DMF, analytical grade, Sigma Aldrich), dimethylsulfoxide (DMSO, analytical grade, VWR Chemicals, Darmstadt, Germany), 4,4′-dinonyl-2,2′-dipyridyl (dNBipy, 97%, Sigma Aldrich), ethyl acetate (EtOAc, analytical grade, Chem Solute, Renningen, Germany), hexane (analytical grade, Fluka, Schwerte, Germany), hydrochloric acid (fuming, Carl Roth), magnesium sulfate (dried, Fisher Scientific, Schwerte, Germany), methanol (MeOH, analytical grade, Fisher Scientific), *N,N,N′,N″,N*″-pentamethyldiethylenetriamine (PMDETA, 98%, Sigma Aldrich), potassium-O-ethyl xanthate (98%, Alfa Aesar), propargyl alcohol (99%, Sigma Aldrich), pyridine (99% extra dry, Acros Organics), sodium hydroxide (NaOH, 98%, Sigma Aldrich), sodium azide (>99.5%, Fluka), sodium bicarbonate (>99%, Fluka), sodium sulfite (97%, Acros Organics), tannic acid (Alfa Aesar), tetrahydrofuran (THF, analytical grade, Fisher Scientific), and triethylamine (99.5%, Sigma Aldrich) were used as received. *N*-Vinylpyrrolidone (VP, 99%, Sigma Aldrich) was dried over anhydrous magnesium sulfate and purified by distillation under reduced pressure. Oligo(ethylene glycol) methyl ether methacrylate (OEGMA, 900 g mol^−1^, Sigma Aldrich) was first dissolved in THF, and then passed over a basic aluminum oxide column (Brockmann I, Sigma Aldrich) and subsequently precipitated in cold hexane, filtered, and dried under high vacuum for 24 h. Millipore water was obtained from an Integra UV plus pure water system by SG Water (Hamburg, Germany). Azido functionalized poly(styrene)-resin, prop-2-yn-1-yl 2-((ethoxycarbonothioyl)thio) propanoate (alkyne-CTA), alkyne end-functionalized PVP (Figure S1), and azide end functionalized P(OEGMA) (Figure S2) were prepared according to the literature [30,36,58]. Spectra/Por dialysis tubes with a molecular weight cut-off of 10,000 were purchased from Spectrum Labs (Los Angeles, CA, USA). 

Procedure for the preparation of DHBC aqueous solutions for DLS investigation: For the DLS measurements, 50.0 mg of DHBC were dissolved in 2.5 g of Millipore water in order to obtain a 2.0 wt.% DHBC solution. Before conducting the DLS measurement, the solution was passed through a 1.2 µm filter. The solutions containing 0.5 wt.% and 0.1 wt.% of DHBC were obtained by diluting the initial 2.0 wt.% DHBC solution with Millipore water accordingly. 

Crosslinking of PVP-b-P(OEGMA) via TA: In order to crosslink the PVP-*b*-P(OEGMA) aggregates, a 0.5 wt.% TA solution was added to a 10.0 wt.% PVP-*b*-P(OEGMA) solution to form a 2.0 wt.% PVP-*b*-P(OEGMA)-TA-solution. Then, the mixture of TA with the polymer was characterized via DLS, cryo-SEM, and AUC. The 0.5 wt.% and 0.1 wt.% solutions were obtained by diluting the 2.0 wt.% solutions accordingly.

Disassembly of PVP-b-P(OEGMA) TA crosslinking via NaOH: In order to induce a disassembly of the PVP-*b*-P(OEGMA) aggregates, first, a crosslinking reaction was applied as reported in previous sections receiving 2 g of a 2.0 wt.% crosslinked DHBC solution. Subsequently, 40 mg of NaOH was added to the solution to obtain a 0.5-m NaOH solution. The solution was stirred for 1 h before it was passed through a 1.2-µm filter followed by its investigation via DLS and cryo-SEM. The 0.5 wt.% and 0.1 wt.% solutions for the DLS investigations were obtained by diluting the 2.0 wt.% solution accordingly.

AUC measurements of PVP-b-P(OEGMA): For the AUC measurements, two stock solutions were used. The 2.0 wt.% stock solution of pure DHBC in Millipore water was diluted further to afford the following polymer concentrations: 2.0 wt.%, 1.0 wt.%, 0.5 wt.%, 0.2 wt.%, 0.1 wt.%, 0.05 wt.%, and 0.03 wt.%. The diluted samples as well as the stock solutions were utilized for sedimentation velocity experiments to investigate the sedimentation behavior of pure DHBC PVP-*b*-P(OEGMA). In the case of TA crosslinked PVP-*b*-P(OEGMA), the stock solution is reported in the respective paragraph.

## 3. Results

### 3.1. Synthesis of PVP-b-P(OEGMA)

The synthesis of PVP-*b*-P(OEGMA) was conducted in two steps according to the literature [59]. First, the individual homopolymers were synthesized (Figures S1 and S2); then, the blocks were coupled via CuAAc (Figure S3). Another option of block copolymer formation would be the utilization of switchable chain transfer agents, as reported recently [60]. Although PVP is a widely used polymer and is produced on a large scale, controlled polymerization is quite challenging [61]. Reversible addition–fragmentation chain transfer polymerization employing *tert*-butylhydroperoxide and sodium sulfite as redox initiators was performed at ambient temperature with an alkyne functionalized chain transfer agent to obtain alkyne end-functionalized PVP (*Ð* = 1.25; *M*_n_ = 34,000 g mol^−1^, determined via SEC utilizing a PMMA calibration). On the other hand, P(OEGMA) was synthesized via atom transfer radical polymerization using 2-azidoethyl 2-bromoisobutyrate (*Ð* = 1.06; *M*_n_ = 21,000 g mol^−1^, determined via SEC utilizing a PMMA calibration). Finally, the alkyne end-functionalized PVP block and the azido end-functionalized P(OEGMA) brush were conjugated via CuAAc in a mixture of water and DMSO according the literature [62]. To ensure full conversion, first, an excess of alkyne terminated PVP was used, whereas after 3 days of reaction, an azido-methyl polystyrene resin was added to the reaction mixture to capture the excess of PVP [30]. The presence of both blocks was verified via ^1^H-NMR (Figure S3b), which shows the characteristic signal of the proton assigned to the triazole at 8.1 ppm, and thus demonstrates the success of the coupling reaction. Moreover, the successful conversion to the desired DHBC could be shown by a clear shift in the SEC molar mass distribution (*Ð* = 1.42; *M*_n_ = 59,000 g mol^−1^, determined via SEC utilizing a PMMA calibration) (Figure S3c).

### 3.2. PVP-b-P(OEGMA) Aggregate Formation

**Figure 1:**
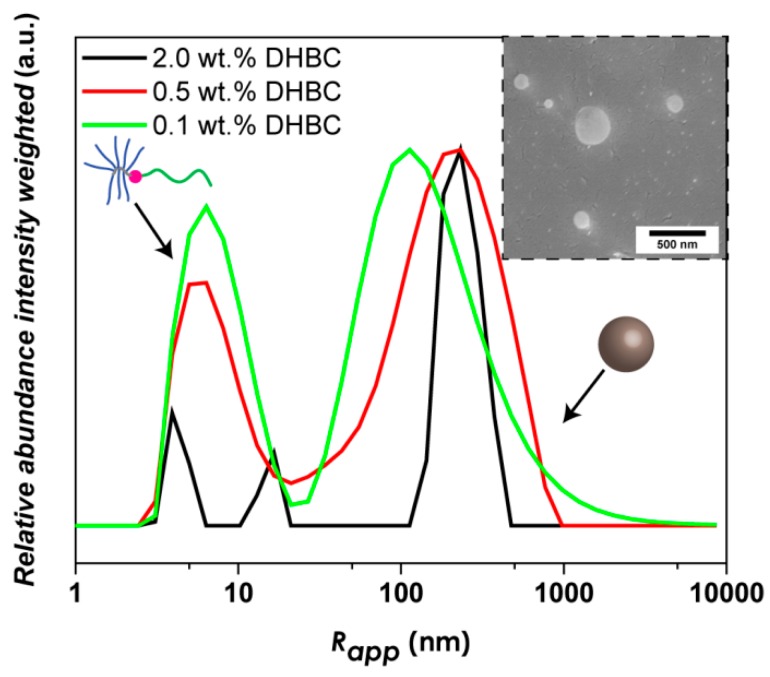
Intensity-weighted size distribution of PVP-*b*-P(OEGMA) in Millipore water measured via dynamic light scattering (DLS) at 25 °C and at concentrations between 0.1–2.0 wt.%. Inset: Cryogenic scanning electron microscopy (cryo-SEM) imaging of PVP-*b*-P(OEGMA) DHBC at a concentration of 2.0 wt.%, indicating existence of larger structures.

After synthesis of the PVP-*b*-P(OEGMA), intended self-assembly in aqueous solution was investigated (Table S1). As known from the literature, the self-assembly of DHBCs in water is strongly dependent on polymer concentration [25]. To study the self-assembly behavior of PVP-*b*-P(OEGMA), DLS was conducted to identify the presence of aggregate formation on the one hand, and, on the other hand, to qualitatively compare the fraction of the unimers to the fraction of aggregates formed as a possible hint toward intended self-assembly. After dissolving PVP-*b*-P(OEGMA) in Millipore water, DLS measurements in intensity mode clearly show the formation of aggregates with apparent hydrodynamic radii in the range of 100 to 250 nm (Figure 1, Table S1), which is supported by cryo-SEM images (Figure 1 and Figure S4). DLS data in intensity mode show not only the existent self-assembly of PVP-*b*-P(OEGMA), but also the strong dependence on concentration in solution. While diluting the solution from 2.0 wt.% to 0.5 wt.%, and finally to 0.1 wt.%, the apparent fraction of the non-assembled polymers increases pronouncedly. This indicates a highly dynamic aggregate formation, depending on concentration. Furthermore, we note that the displayed intensity-weighted size distributions and obtained abundances are overestimating larger structures, which is a well-known issue of DLS analysis in solution. Thus, only a small fraction of self-assembled structures exists in solution, which can be nonetheless observed clearly in the intensity distribution. Due to the small amount of aggregates in solution, in the further course of the project, ways to improve the situation were studied. Hence, non-covalent crosslinking and stabilization of the structures was performed subsequently to investigate whether the equilibrium of aggregates and unimers can be shifted toward aggregates.

Compared to the analogous linear–linear block copolymer PVP-*b*-PEO [41], a significant increase in the apparent self-assembly efficiency, as probed by intensity-weighted DLS, can be observed (Figure 1). While in the case of a 2.5 wt.% linear–linear DHBC, DLS shows the presence of double the free polymer than of self-assembled aggregates [41]. Changing the polymer architecture to linear-brush DHBC shows a three times higher presence of aggregates than unimers at 2.0 wt.% in the DLS intensity. Therefore, a significant effect of polymer architecture on the properties can be concluded. This highlights the importance of structure–property relationships in DHBC self-assembly and aggregate formation.

### 3.3. Crosslinking of DHBC Aggregates via Tannic Acid 

**Figure 2:**
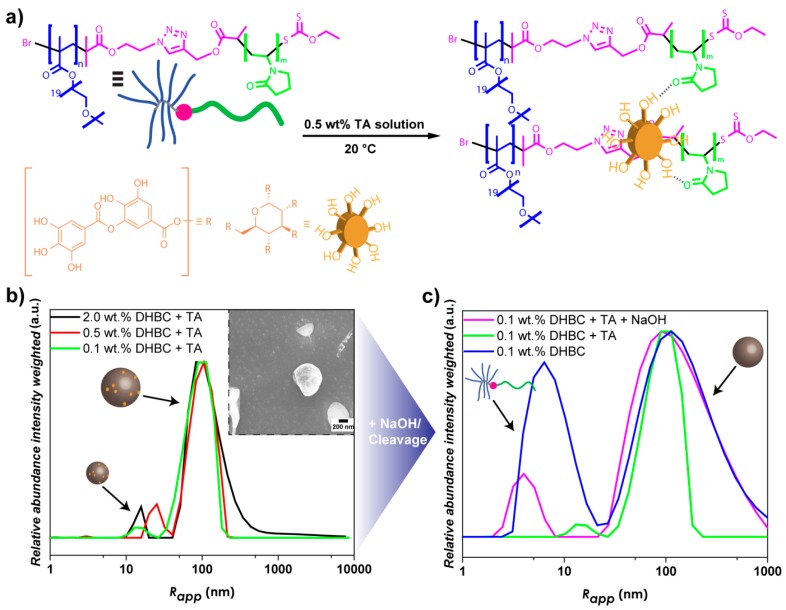
(**a**) Schematic overview of the non-covalent crosslinking reaction of PVP-*b*-P(OEGMA) with tannic acid (TA). (**b**) Intensity-weighted aggregate size distribution of PVP-*b*-P(OEGMA) after crosslinking with TA in Millipore water, measured via DLS at 25 °C (Inset: Cryo-SEM image of PVP-*b*-P(OEGMA) at a concentration of 2.0 wt.% after crosslinking with TA). (**c**) Intensity-weighted size distributions of PVP-*b*-P(OEGMA) measured via DLS at 25 °C before and after the non-covalent crosslinking procedure via TA, and the subsequent disassembly of aggregates via the addition of NaOH at 2.0 wt.%.

In order to shift the equilibrium between unimers and aggregates toward aggregate formation by an enhanced self-assembly stability, the aggregates were attempted to be crosslinked with TA (Figure 2a). TA is well-known for its supramolecular complexation with PVP polymers [63]. Crosslinking experiments via hydrogen bonding were conducted by diluting a 10 wt.% PVP-*b*-P(OEGMA) solution to 2.0 wt.% via a 0.5 wt.% aqueous TA solution (see SI for details). The crosslinking reaction takes place instantly, and is visually observed by a slight turbidity of the solution. Noteworthy, adding a TA solution of higher concentration (0.6 wt.%) resulted in sedimentation of the polymer–TA aggregates. This shows the strong supramolecular complexation of TA with the PVP of DHBC, while a diluted TA solution (0.3 wt.%) does not show a significant promotion of large aggregate formation. Rather, a disassembly is observed due to dilution after addition of the TA solution. Thus, a ratio of 1:7 (PVP-*b*-P(OEGMA):TA) indicates a strong non-covalent crosslinking. While diluting, the phenolic hydroxide groups undergo strong hydrogen bonding with the peptide-like functional groups of PVP located within the DHBC. Complex formation appears not only fast, but also appears stable up to the boiling point of water. This may qualify for a simple and straightforward technique to stabilize the DHBC aggregates. Non-covalent crosslinking could also be followed via Fourier transform infrared (FT-IR) spectroscopy (Figure S5). A clear broadening of the OH-stretching vibration of TA after crosslinking indicates the existence of hydrogen bonds stabilizing aggregate formation.

After the successful crosslinking of PVP-*b*-P(OEGMA) via TA, significant changes of the self-assembly could be observed via DLS in intensity-weighted size distributions (Figure 2b). Most notably, the intensity-weighted abundance of unimers decreases after the addition of TA. The self-assembly process is a highly dynamic equilibrium which leads to an ongoing self-assembly/disassembly of the unimers toward aggregates, as well as the possible exchange of unimers in aggregates by unimers in solution. The addition of TA to the DHBC solution appears to “freeze” the existent self-assembled aggregates via hydrogen bonding interactions. Moreover, the formation of smaller aggregates in size is observed, which were apparently absent before the addition of TA. The consolidated self-assembly after TA addition and stabilization of the formed aggregates can be observed in two different manners. First, prior to crosslinking, aggregate stability shows a strong concentration dependency resulting in a pronounced unimer fraction when diluted, which appears to not be the case after non-covalent crosslinking. For example, at 0.1 wt.% of polymer, almost no unimer fraction can be detected after crosslinking, but the DHBC solution shows the presence of free polymer with an apparent abundance of up to 84% according to the intensity-weighted particle size distribution. Second, the stabilizing effect of TA can also be considered from the concentration dependency of the apparent aggregate size. Before crosslinking, the particle size decreases with decreasing concentration; a similar dynamic behavior cannot be observed after crosslinking. On first sight, this may lead to a stabilized aggregate size upon dilution, indicating the non-covalent crosslinking ability of TA. Furthermore, the cryo-SEM imaging appears to underline the intensity-weighted DLS results by the presence of aggregates with apparent overall sizes around 300–400 nm (Figure 2b and Figure S6). As well, small aggregates with a hydrodynamic radius of around 15 nm can be observed, which may correspond to the smaller aggregates formed after crosslinking.

### 3.4. Base-Induced Disassembly of Crosslinked PVP-b-P(OEGMA) Aggregates

As TA is building hydrogen bonds with the lactam group within PVP, a change in the pH value via the addition of base leads to the suppression of such interactions, as the deprotonated TA cannot undergo hydrogen bonding with the lactam groups in PVP [64]. Nevertheless, it has been shown that the PVP–TA complex can be stable up to a pH of 10 [65]. TA crosslinked PVP-*b*-P(OEGMA) aggregates could be disassembled by the addition of sodium hydroxide (NaOH), as illustrated via DLS (Figure 2c and Figure S7). The self-assembly behavior of pure and TA crosslinked aggregates were compared to the self-assembly behavior of DHBC aggregates that were first crosslinked via TA and afterwards disassembled via the addition of NaOH. The samples were diluted from their initial polymer concentration (2.0 wt.%) to 0.5 wt.% and 0.1 wt.% in order to observe the re-introduced dynamics of the aggregates (Figure S7). Notably, no aggregates of pure TA were observed via DLS after deprotonation with NaOH.

While the solution of non-covalently crosslinked aggregates shows decreased amounts of unimers, and only small aggregates with a hydrodynamic radius of around 15 nm as well as large aggregates with a radius in the range of 100 nm, the addition of NaOH led to the disassembly of the small aggregates and the appearance of unimers. Therefore, the increase in pH value directly resulted in a breakdown of parts of the hydrogen bonds between TA and the PVP block. The existence of some reversibility of the crosslinking can be observed particularly after diluting the sample from the initial 2.0 wt.% to 0.5 wt.% (Figure S7) and 0.1 wt.% (Figure 2c). While the crosslinked DHBC does not show a major change in the self-assembly during dilution (Figure 2b), pure DHBC shows substantial changes as the unimer fraction is increased after each dilution step. Such dynamics are typical for non-crosslinked aggregates, as the concentration directly affects the ratio of unimers and aggregates. Aggregates that were first crosslinked via TA and afterwards treated with NaOH show a similar behavior as the unimer fraction increased with each dilution step. However, the non-covalent crosslinking is not fully reversible, as the initial fraction of unimers in pure DHBC is significantly higher than the unimer fraction after NaOH treatment (Figure 2c).

### 3.5. PVP-b-P(OEGMA) Aggregate Characterization via Analytical Ultracentrifugation

**Figure 3:**
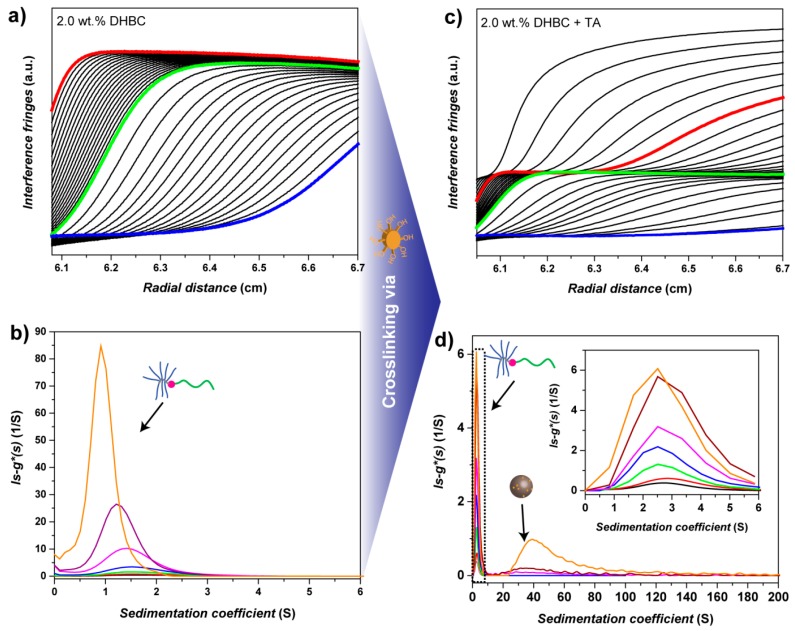
(**a**) Selected sedimentation velocity profiles of aqueous PVP-*b*-P(OEGMA) solution at 2.0 wt.% concentration with highlighted profiles more close to the beginning of the experiment (red), at an intermediate timescale (green), and close to the end (blue). (**b**) Sedimentation coefficient distribution, ls-g*(s), of PVP-*b*-P(OEGMA) with varying concentrations (0.03 wt.% (black), 0.05 wt.% (red), 0.1 wt.% (green), 0.2 wt.% (blue), 0.5 wt.% (magenta), 1.0 wt.% (brown), 2.0 wt.% (orange)). (**c**) Sedimentation velocity profiles of aqueous PVP-*b*-P(OEGMA) solution after the non-covalent crosslinking with TA was performed at 2.0 wt.% polymer concentration with highlighted profiles as in (**a**). (**d**) Sedimentation coefficient distribution, ls-g*(s) of PVP-*b*-P(OEGMA) after the non-covalent crosslinking reaction with various dilutions (0.03 wt.% (black), 0.05 wt.% (red), 0.1 wt.% (green), 0.2 wt.% (blue), 0.5 wt.% (magenta), 1.0 wt.% (brown), 2.0 wt.% (orange)), inset: magnification for the range of low sedimentation coefficients.

Another solution-based analytical method to study colloidal structures is analytical ultracentrifugation (AUC). AUC is a well-known method that enables following the sedimentation of colloidal structures in the dispersed or dissolved state. The method can provide absolute information of the colloid distribution based on typically employed concentration-sensitive detection. In recent reports, AUC failed in the identification of aggregates that were observed by the utilization of other analytical methods in studying pure DHBC and its aggregates so far [29,35]. In the present study, we utilized concentration-sensitive RI detection for the observation of sedimentation boundaries in sedimentation velocity experiments. Figure 3a clearly shows the apparent observation of a single boundary of aqueous PVP-*b*-P(OEGMA) solutions at 2 wt.% concentration. Notwithstanding, after non-covalent crosslinking, aggregates of DHBC were clearly identified via AUC at the same concentration (Figure 3c).

**Figure 4:**
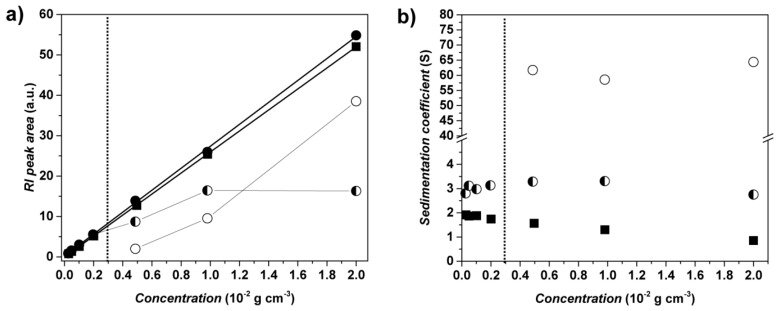
(**a**) Total refractive index (RI) intensities from peak areas of differential sedimentation coefficient distributions, ls-g*(s), for DHBC at varying concentrations (filled squares: without TA; filled circles: with TA) as well as RI peak areas of the population sedimenting at lower sedimentation coefficients (half-filled circles, see Figure 3d) and the abundant larger species (empty circles, see Figure 3d) appearing when non-covalent crosslinking with TA is performed. (**b**) Concentration dependence of the weight-average sedimentation coefficients (filled squares: without TA) and the two populations observed by non-covalent crosslinking with TA, sedimenting with lower sedimentation coefficients (half-filled circles, see Figure 3d) and higher sedimentation coefficients (empty circles, see Figure 3d).

Again, the sedimentation velocity profiles of non-crosslinked PVP-*b*-P(OEGMA) has a one-step profile, which roughly corresponds to a single population of species in solution (Figure 3a). Surprisingly, we found the absence of larger structures. This could be explained by their overrepresentation in DLS-derived intensity-weighted size distributions that over-express the abundance of larger structures in solution. In most of the recent studies, intensity-weighted DLS investigations have been utilized as well, demonstrating the necessity of other in-depth studies to reconcile the dynamics in solution; most prominently, analytical ultracentrifugation failed [29,35]. The present study indicates the existence of aggregates under certain conditions. Furthermore, considering the timescale of the sedimentation velocity experiments, together with the dynamic nature and the weak hydrophilic–hydrophilic interactions of the aggregates, may make the observation of a distinct population abundance in AUC with RI detection impossible. Interestingly, decreased concentrations of the polymer (Figure 3b and Figure 4a) led to a shift of the sedimentation coefficient distribution toward larger values, which is a strong indication of non-ideality observed at these rather high concentrations of the polymer in solution (Figure 3b and Figure 4b) [66,67]. However, the sedimentation profiles of non-covalently crosslinked aggregates with TA show a step in the sedimentation velocity profiles. This could only be the result of two clearly distinct species moving at distinctly different speeds in the centrifugal field (Figure 3c). The apparent sedimentation coefficient distribution of suspected “unimers” and the non-covalently crosslinked aggregates is shown in Figure 3d. The first population at an apparent sedimentation coefficient of 2.5 S (signal weight average), and being largely independent of concentration appears to be the result of PVP-*b*-P(OEGMA) “unimers” (Figure 4b). The apparent second population located at ca. 60 S (signal weight average) indicates the existence of non-covalently crosslinked aggregates formed by the DHBC and TA (Figure 3d and Figure 4b). In contrast to Figure 3b, the sedimentation coefficient distributions of species located around 3 S shows less concentration dependency (as seen by the magnification in the inset of Figure 3d and in Figure 4b). The population of species observed at higher sedimentation coefficients indicates the existence of aggregates with increased abundance at increased concentrations of polymer in solution (Figure 4a, empty circles versus half-filled circles). Interestingly, the total amount of material observed in sedimentation velocity experiments appears readily similar (Figure 4a, filled circles and filled squares), indicating the overall correctness of the mass balance.

Apparently, comparison of the sedimentation velocity results via AUC with DLS-derived intensity-weighted size distributions displays a modified picture of non-covalently crosslinked structures, although both methods show the occurrence of DHBC unimers and aggregates. Experiments at varying concentrations show that below 0.3 wt.%, no distinction between smaller and larger species appears possible (Figure 4) in AUC experiments. However, above 0.3 wt.%, distinction is clearly possible, with the aggregates increasing in abundance and the smaller colloid fraction decreasing in abundance. The overall mass balance of material by summing both signals appears, on first sight, only slightly affected, if at all (Figure 4a). Additionally, the apparent sedimentation velocity of the crosslinked aggregates does not show a pronounced effect on concentration (Figure 4b, empty circles), which clearly underlines their persistence, once observed in AUC. Translation of the signal (weight) average sedimentation coefficients to an apparent size is possible via the hydrodynamic equivalent sphere concept $d_{h}=3\sqrt{2}\sqrt{\left[s\right]\upsilon }$ with

$$\left[s]=s\eta /(1-\upsilon \rho_{0}\right)$$

with $\rho_{0}$ being the solvent density, η being the solvent viscosity, and υ being the partial specific volume of the objects in solution. υ was determined via densimetry, resulting in values of $\upsilon =0.81\;{\mathrm{cm}}^{3}g^{-1}$ for the polymeric system without TA and $\upsilon =0.72\;{\mathrm{cm}}^{3}g^{-1}$ for the system containing TA (see Figure S8). Utilization of these values leads to an apparent hydrodynamic radius of 2.5 nm for the unimers at 2 wt.% polymer without TA, 2.4 nm for the unimers, and 11 nm for the aggregates of solutions containing TA. Here, we assume solid spheres without hydration and modified friction when comparing to an ideal sphere under solution conditions at the respective concentrations. We note that the solution conditions are far away from high dilution, which is required for correct size estimations. Notwithstanding, AUC supports the findings from DLS in terms of the presence of unimers and aggregates that are similar in size when considering the volume-weighted DLS distribution (Figure S9). As indicated by DLS and cryo-SEM, the small abundance of large aggregates (in size around 100 nm) occur only in low concentrations, and thus are difficult to find in AUC measurements. This aspect is not surprising concerning the physical methodology used for deriving apparent hydrodynamic sizes in non-ideal solutions based on concentration-sensitive detection. The relatively small aggregates may also be gauged from the cryo-SEM images (e.g., Figure 2b inset and Figures S4 and S6 at high magnification).

## 4. Conclusions

In the present work, we could show an apparent improvement in the self-assembly process of double hydrophilic block copolymers in aqueous solution by non-covalent crosslinking with TA and by utilizing a linear-brush macromolecular double hydrophilic architecture. The improved self-assembly behavior of PVP-*b*-P(OEGMA) and its concentration dependency could be shown by DLS-derived intensity-weighted size distributions (apparent hydrodynamic radii ≈ 100–120 nm). Moreover, the DHBC assemblies could be non-covalently crosslinked via TA, which led to the additional formation of small aggregates (apparent hydrodynamic radii ≈ 15 nm). The crosslinking improved the self-assembly and apparently stabilized the aggregates upon dilution, diminishing the pronounced concentration dependency of self-assembly of the initial DHBC polymer. The presence of the aggregates could be observed via cryo-SEM before and after non-covalent crosslinking. Furthermore, particularly, the DLS results could be supported with AUC results, showing the existence of both aggregates and unimers, which was practically impossible in recent studies. Although the quantitative agreement between analytical methods is difficult to grasp momentarily, our study as well hints toward the necessity of the use of complementary analytical methods in solution to obtain a comprehensive picture of non-covalent assemblies, as the different methods provide an orthogonal clue about the physical chemistry in solution.

## Supplementary Materials

The Supplementary Materials are available online at https://www.mdpi.com/2079-4991/9/5/662/s1.
