# Supplementary material for: Tannic Acid-Mediated Aggregate Stabilization of Poly(N-vinylpyrrolidone)-b-poly(oligo (ethylene glycol) methyl ether methacrylate) Double Hydrophilic Block Copolymers
Source: Nanomaterials (Basel). 2019 Apr 26;9(5):662. doi: 10.3390/nano9050662 (PMC6566864; doi:10.3390/nano9050662)
Supplement: Supplementary file 1 [file nanomaterials-09-00662-s001.pdf]

Supporting Information

Tannic Acid-Mediated Aggregate Stabilization of Poly(*N*-vinylpyrrolidone)-*b*-poly(oligo (ethylene glycol) methyl ether methacrylate) Double Hydrophilic Block Copolymers

Noah Al Nakeeb [1], Ivo Nischang [2,3,*] and Bernhard V.K.J. Schmidt [1,*]

[1] Max-Planck Institute of Colloids and Interfaces; Department of Colloid Chemistry, Am Mühlenberg 1, 14476 Potsdam, Germany; noah.alnakeeb@gmail.com
[2] Laboratory of Organic and Macromolecular Chemistry (IOMC), Friedrich Schiller University Jena, Humboldtstraße 10, 07743 Jena, Germany
[3] Jena Center for Soft Matter (JCSM), Friedrich Schiller University Jena, Philosophenweg 7, 07743 Jena, Germany
* Correspondence: bernhard.schmidt@mpikg.mpg.de (B.V.K.J.S.); ivo.nischang@uni-jena.de (I.N.)

Additional Synthetic procedures

Prop-2-yn-1-yl 2-bromopropanoate[1]

In a dry, argon purged 250 mL Schlenk tube, propargyl alcohol (3.94 g, 4.1 mL, 70.2 mmol, 1.0 eq.) and triethylamine (9.95 g, 13.6 mL, 98.3 mmol, 1.4 eq.) were dissolved in dry THF (150 mL). The reaction mixture was cooled to 0 °C and 2-bromopropionyl bromide (18.19 g, 8.83 mL, 84.24 mmol, 1.2 eq.) was added slowly, dropwise to the reaction mixture. The reaction mixture was allowed to adjust to ambient temperature and stirred for five hours at ambient temperature. The formed salt was filtered off and the organic phase was subsequently washed with 2 M HCl solution (3 × 10 mL), deionized water (3 × 30 mL), saturated $NaHCO_3$ solution (3 × 10 mL), and deionized water (3 × 30 mL). The organic phase was dried over anhydrous $MgSO_4$ and the solvent was removed under reduced pressure. The crude product was purified via vacuum distillation (98 °C, 37 mbar) to afford prop-2-yn-1-yl 2-bromopropanoate (8.00 g, 42.11 mmol, 60% yield) as slightly yellow liquid.

^{1}H NMR (400 MHz, $CDCl_3$ *δ*:) 4.76 (dd, ^1J = 3.8 Hz, ^4J = 2.5 Hz 2H, *CH_2*), 4.40 (q, ^3J = 6.9 Hz, 1H, *CH*), 2.52 (t, ^4J = 2.5 Hz, 1H, *alkyne-H*), 1.84 (d, ^3J = 6.9 Hz, 3H, *CH_3*).

Prop-2-yn-1-yl 2-((ethoxycarbonothioyl)thio) propanoate (alkyne-CTA)[1]

In a dry, argon purged 250 mL Schlenk flask, prop-2-yn-1-yl 2-bromopropanoate (3.0 g, 15.79 mmol, 1.0 eq.) was dissolved in dry THF (200 mL). Potassium *O*-ethyl xanthate (25.78 g, 157.93 mmol, 10.0 eq.) was added to the solution under argon flow. The reaction

mixture was stirred over night at ambient temperature. The formed salt and the excess of potassium *O*-ethyl xanthate was filtered off and the organic phase was washed with deionized water (4 × 75 mL) and dried over anhydrous $MgSO_4$. The solvent was evaporated to afford prop-2-yn-1-yl 2-((ethoxycarbonothioyl)thio) propanoate (3.15 g, 13.55 mmol, 86% recovery) as a yellow oil.

^{1}H NMR (400 MHz, $CDCl_3$ δ:) 4.74 (d, ^4J = 2.5 Hz 2H, *CH₂*), 4.61 (q, ^3J = 7.1 Hz, 2H, CH_2O), 4.41 (q, ^3J = 7.4 Hz, 1H, *CH*), 2.52 (t, ^4J = 2.5 Hz, 1H, *alkyne-H*), 1.58 (d, ^3J = 7.4 Hz, 3H, CH_3) 1.42 (t, ^3J = 7.1 Hz, 3H, CH_3).

Synthesis of azidomethyl polystyrene resin[2]

In a dry, argon purged 100 mL round bottom Schlenk tube, chloromethyl polystyrene resin (10.0 g, 24.0 mmol, 1.0 eq.) was dissolved in dry DMSO (50 mL). Sodium iodide (10.8 g, 72.0 mmol, 3.0 eq.) and sodium azide (15.6 g, 240.0 mmol, 10.0 eq.) were added and the reaction mixture was moderately stirred for 48 hours at 80 °C. The afforded resin was filtered over a glass frit (pore size 3; 16-40µm) and alternately washed with DCM (6× 30 mL) and MeOH (6 × 30 mL). The purified resin was finally washed with diethyl ether (30 mL) and dried under vacuum to afford azidomethyl polystyrene resin (9.25 g, 22.2 mmol, 93% recovery) as a white solid. FT-IR ($\tilde{\nu}$ in cm^{-1}): 3120, 2855, 2089 (N_3), 1509, 1450, 750, 697.

Synthesis of PVP_{34k}

In a dry, argon purged 25 mL Schlenk tube, prop-2-yn-1-yl 2-((ethoxycarbonothioyl)thio)propanoate (0.0175 g, 0.075 mmol, 1.0 eq.) was dissolved in deionized water (3.36 mL). VP (4.17 g, 37.5 mmol, 500 eq.) and *t*-BuOOH solution (2.9 mg of 70 wt.% solution, 0.0225 mmol, 0.3 eq.) were added to the solution. Then the mixture was degassed via three freeze, pump, and thaw cycles, followed by a last freeze and purging with argon. While purging with argon, sodium sulfite (3.8 mg, 0.03 mmol, 0.2 eq.) was added. The reaction mixture was then immersed in an oil bath at 25 °C. After 3 h, the polymerization was quenched with liquid nitrogen and exposed to air. Water was removed via reduced pressure and the crude polymer was dissolved in a small amount of MeOH. The mixture was precipitated twice into cold diethyl ether to afford alkyne terminated PVP (PVP-alkyne) as a white powder. (Yield 2.10 g, 76% recovery, $M_{n,SEC}$ = 34 000 g mol^{-1} (PMMA equivalents in NMP), $Đ$ = 1.25).

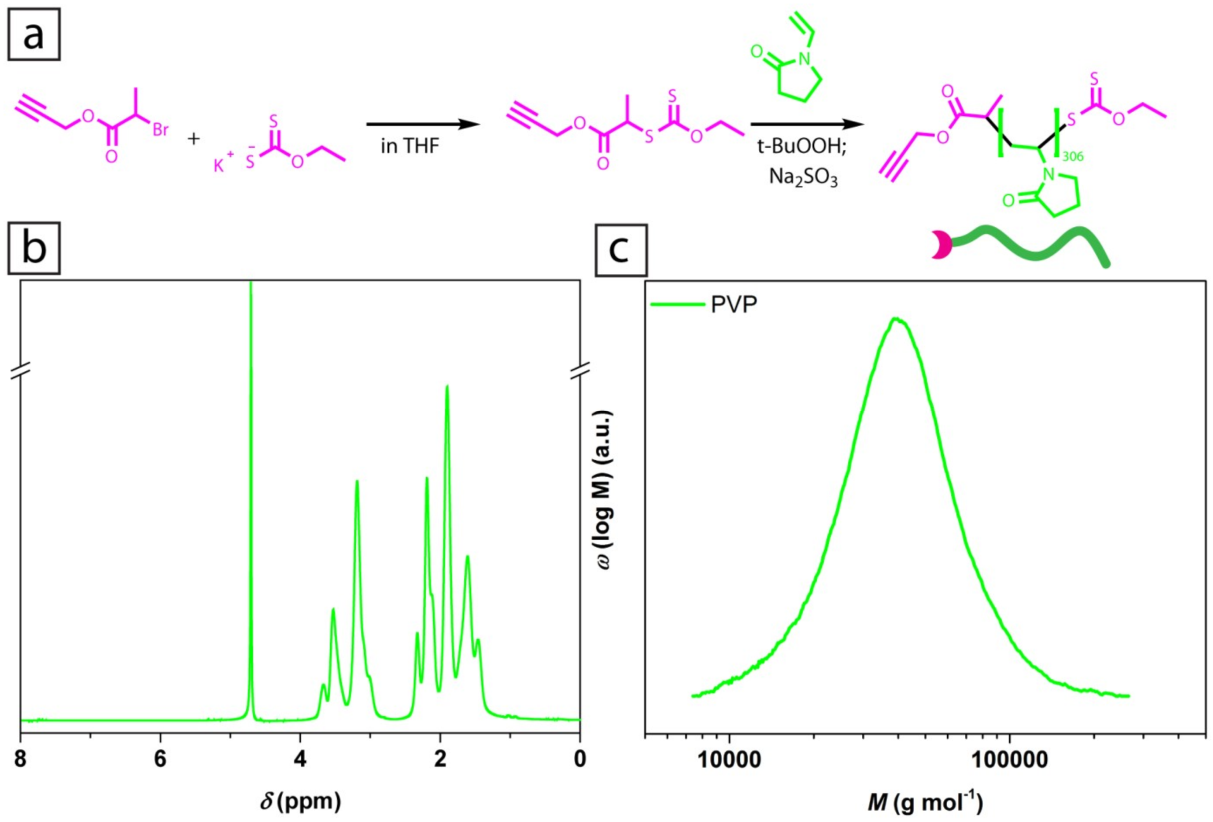

Figure S1. (a) RAFT polymerization of *N*-vinylpyrrolidone via an alkyne functionalized chain transfer agent; (b) ^{1}H-NMR spectrum of PVP_{34k}-alkyne polymer block at 400 MHz in D_2O; (c) molar mass distribution of the corresponding linear PVP-alkyne polymer determined via SEC in NMP using PMMA calibration.

Synthesis of $POEGMA_{21k}$

A stirring bar, 2-azidoethyl 2-bromoisobutyrate (0.047 g, 0.2 mmol, 1 eq), dNbpy (0.16 g, 0.4 mmol, 2 eq), destabilized OEGMA (4.75 g, 5 mmol, 20 eq), and toluene (11 mL, 9.5 g) were placed in a Schlenk tube. The tube was first sealed with a septum and then the mixture was degassed via three freeze, pump, and thaw cycles, followed by a last freeze and purging with argon. While purging with argon, CuBr (0.028 mg, 0.2 mmol, 1 eq) was added to the frozen mixture and the Schlenk tube was purged with argon until the frozen solution thawed and the solution color changed to brown, indicating the formation of the copper-complex. Subsequently, the reaction mixture was placed in an oil bath and stirred at 60 °C for 7 hours. The reaction was cooled to ambient temperature and the reaction was stopped by removing the septum allowing oxygen to enter. The residue was dissolved in THF and passed through a short column filled with neutral aluminum oxide. The resulting solution was concentrated and the polymers precipitated in cold hexane. Subsequently, the solution was filtered and was dialyzed against water (mass cut-off: 10 kDa) for three days. Subsequently, the solution was

lyophilized to afford POEGMA as a colorless solid (Yield 1.90 g, 53% recovery, $M_{n,SEC}$ = 21000 g mol^{-1} (PMMA equivalents in NMP), $Đ$= 1.06).

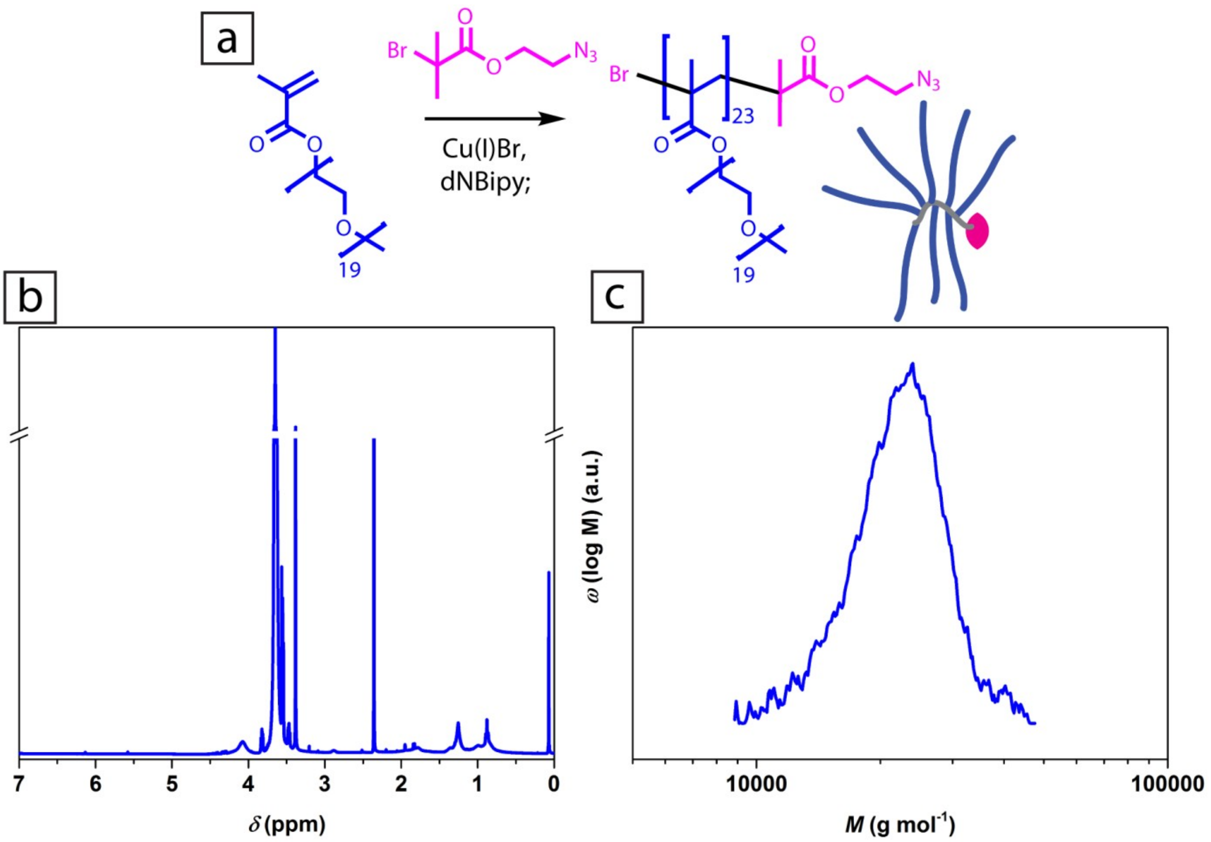

Figure S2. (a) The ATRP of OEGMA to obtain the corresponding P(OEGMA)$_{21k}$-brush polymer block; (b) ^{1}H-NMR spectrum of P(OEGMA)$_{21k}$ polymer block at 400 MHz in DMSO-d$_6$; (c) molar mass distribution of the corresponding P(OEGMA)$_{13k}$-brush polymer block determined via SEC in NMP using PMMA calibration.

Synthesis of PVP$_{34k}$-*b*-POEGMA$_{21k}$

In a dry, argon purged 25 mL round bottom Schlenk flask, alkyne end functionalized PVP (1.63 g, 0.048 mmol, 1.2 eq.) was dissolved in deionized water (10.0 mL). $CuSO_4$ (4.2 mg, 26.0 µmol, 0.65 eq.) and DMSO (10.0 mL) were added to the solution. Azide end functionalized POEGMA (0.84 g, 0.04 mmol, 1.0 eq.) and PMDETA (0.01 g, 0.06 mmol, 1.5 eq.) was dissolved in DMSO (2.0 mL) and added to the reaction mixture. Finally, ascorbic acid (14.0 mg, 0.08 mmol, 2.0 eq.) was added twice, once directly at the beginning of the reaction, and also after 24 hours. The reaction mixture was stirred at ambient temperature for 48 hours. Azido functionalized PS-Resin (21.3 mg, 0.048 mmol) and ascorbic acid (14.0 mg, 0.08 mmol, 2.0 eq.) were added and the reaction mixture was stirred for an additional 48 h. The resin was filtered off and the solution was dialyzed against deionized water for three days

followed by lyophilization to afford PVP_{34k}-*b*-$POEGMA_{21k}$ (1.75 g, 69% recovery M_n = 59000 g·mol^{-1}, PMMA standard in NMP, $Đ$= 1.42) as a white powder.

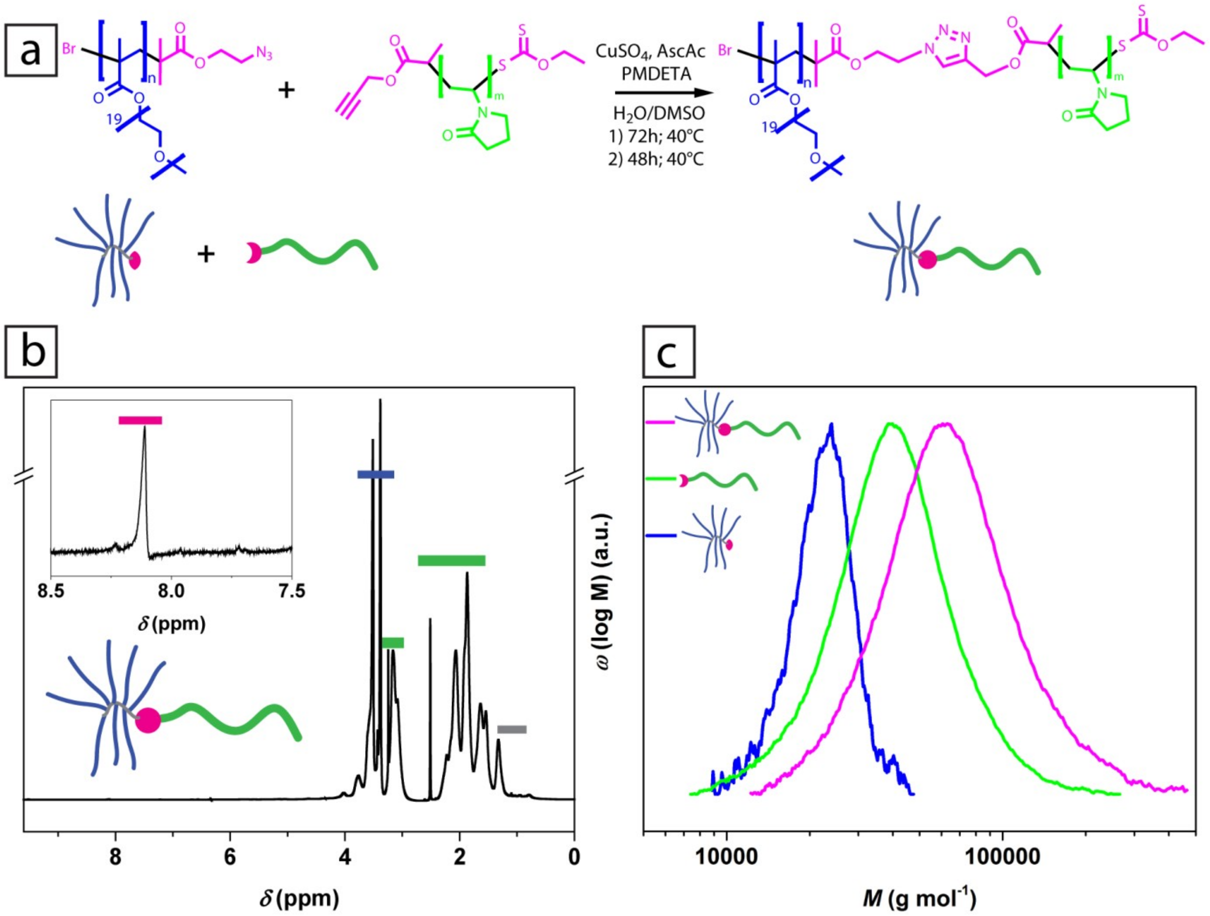

Figure S3. (a) The CuAAC of the P(OEGMA)-brush polymer and the alkyne terminated PVP to afford the PVP-*b*-P(OEGMA) block copolymer. (b) A representative ^{1}H-NMR spectrum of the corresponding PVP_{34k}-*b*-$P(OEGMA)_{21k}$ block copolymer recorded at 400 MHz in DMSO-d_6 (signals from PVP in green, signals from P(OEGMA) in blue, signals from the backbone in grey, signals from the triazole in pink). (c) Corresponding molar mass distributions determined via SEC in NMP using a PMMA calibration.

Table S1. DLS results of PVP-*b*-P(OEGMA) and comparison to the literature.

Sample	*Conc.* (wt.%)	R_{app} 1st peak (nm)	R_{app} 2nd peak (nm)
PVP-*b*-PEO[40]	2.5	5	250
PVP_{34k}-*b*-$P(OEGMA)_{21k}$	2.0	4 /17	232
PVP_{34k}-*b*-$P(OEGMA)_{21k}$	0.5	6	232
PVP_{34k}-*b*-$P(OEGMA)_{21k}$	0.1	6	113

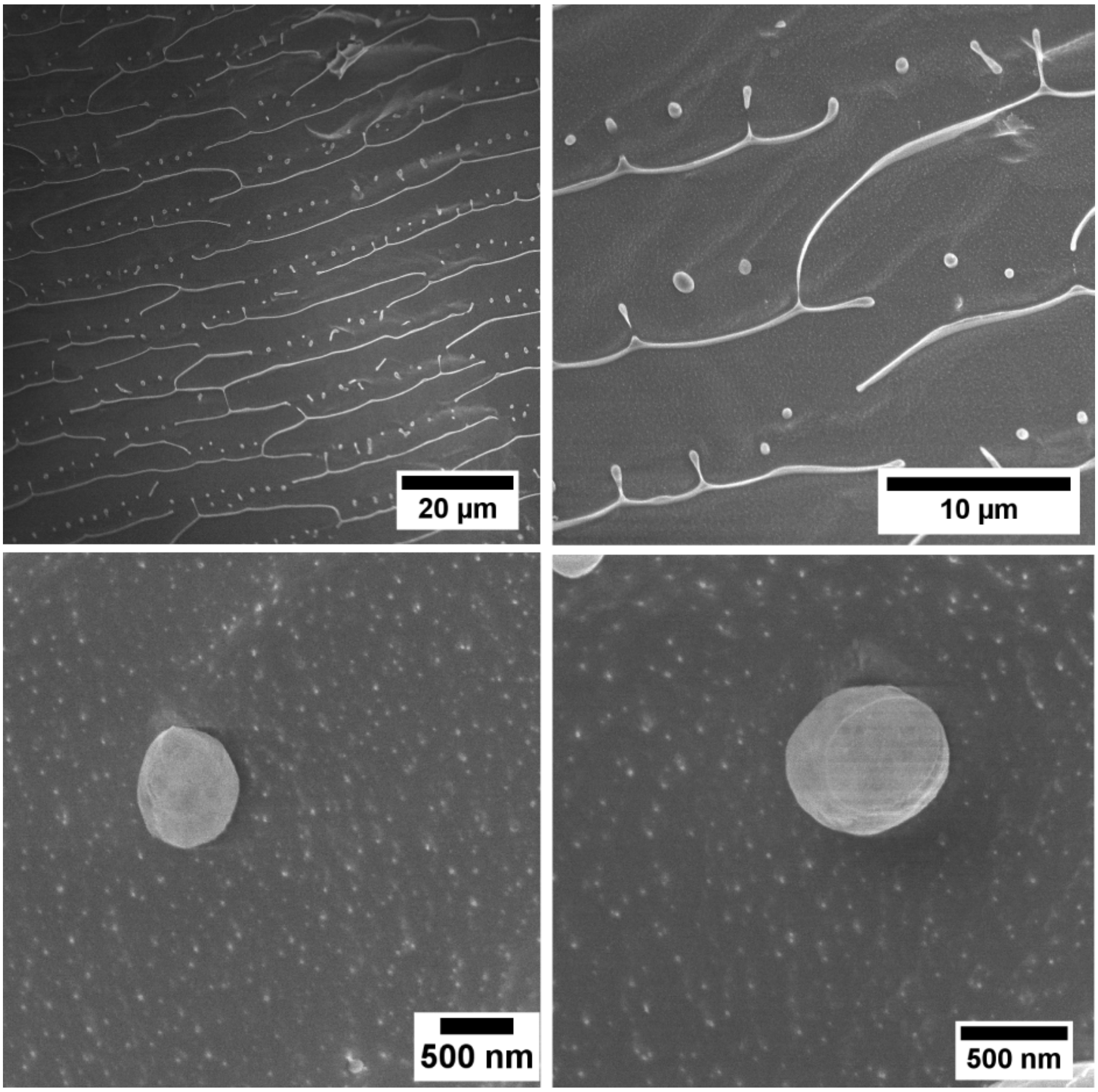

Figure S4. Cryo-SEM imaging of PVP-*b*-P(OEGMA) at a concentration of 2.0 wt.%.

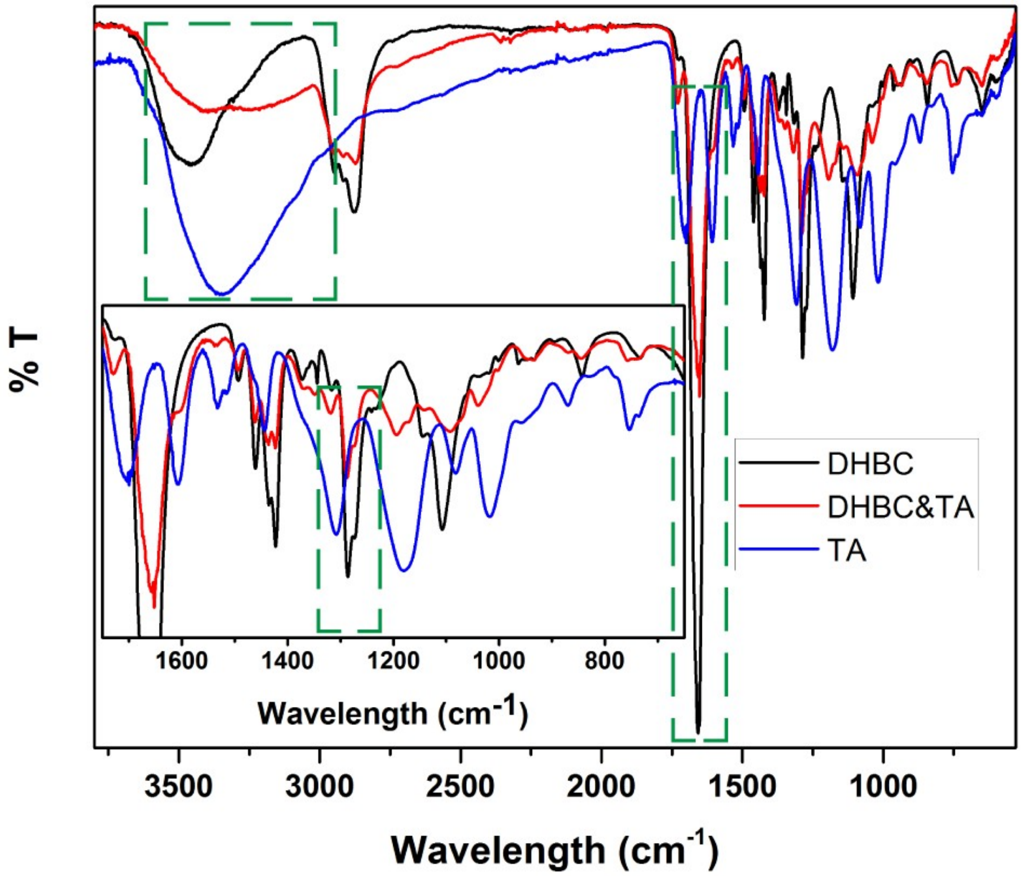

Figure S5. FTIR spectrum of PVP-*b*-P(OEGMA) (black) crosslinked PVP-*b*-P(OEGMA) via TA (red) and TA (blue).

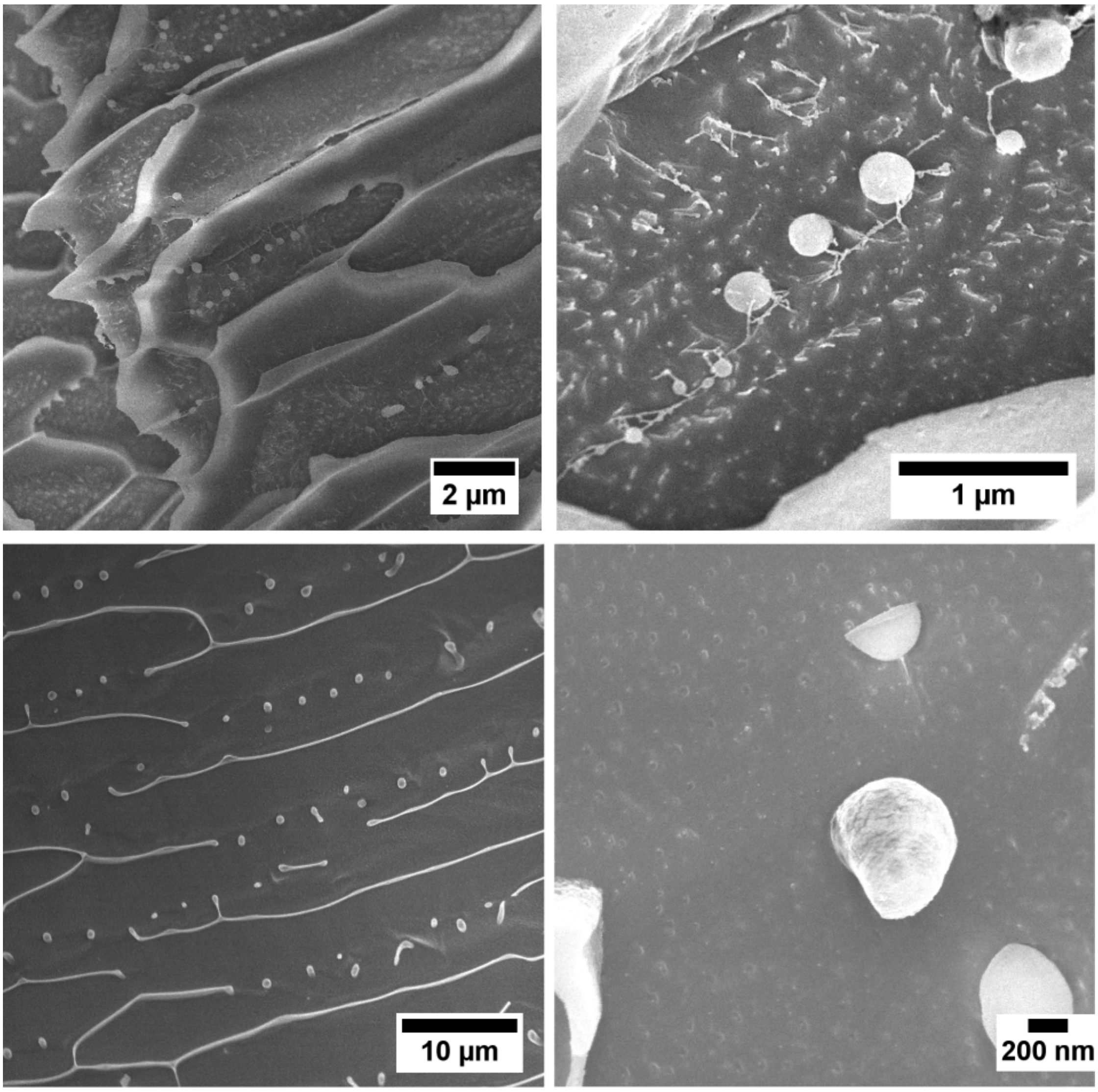

Figure S6. Cryo-SEM imaging of PVP-*b*-P(OEGMA) at a concentration of 2.0 wt.% after crosslinking with TA.

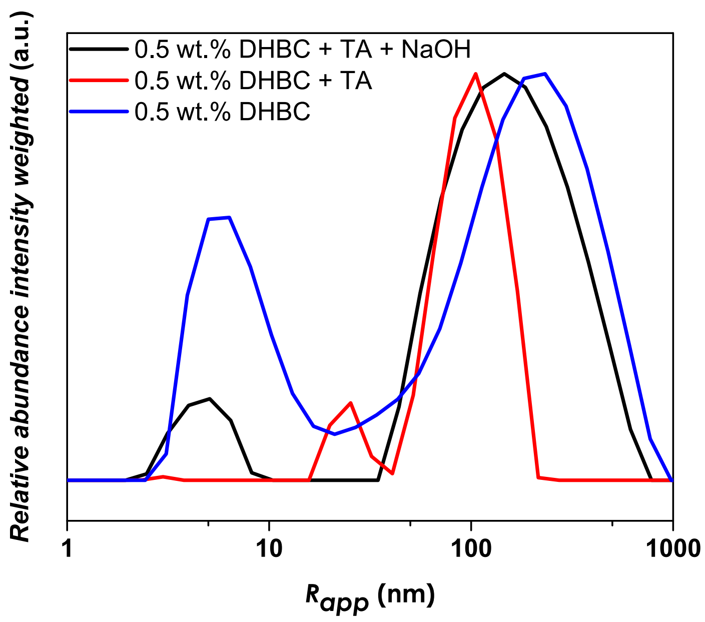

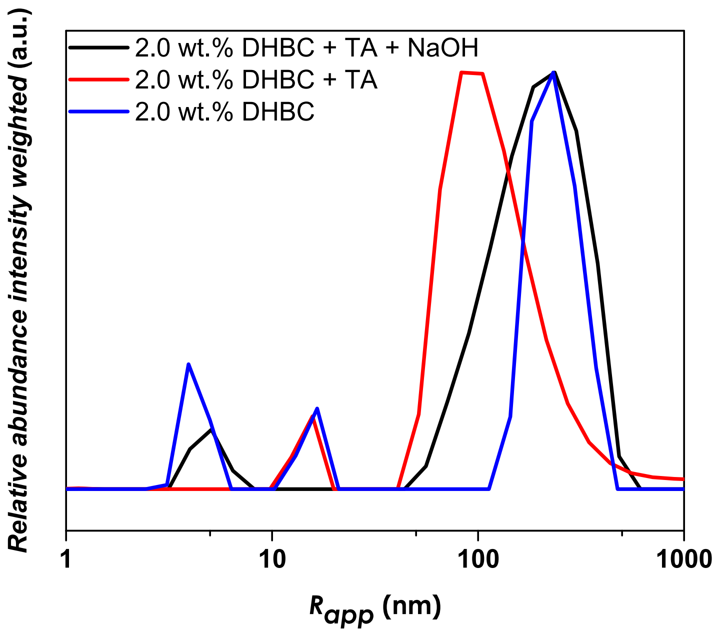

Figure S7. Intensity-weighted size distributions of PVP-*b*-P(OEGMA) measured via DLS at 25 °C before and after the crosslinking procedure via TA and the subsequent disassembly process via the addition of NaOH at 2.0 wt.%.

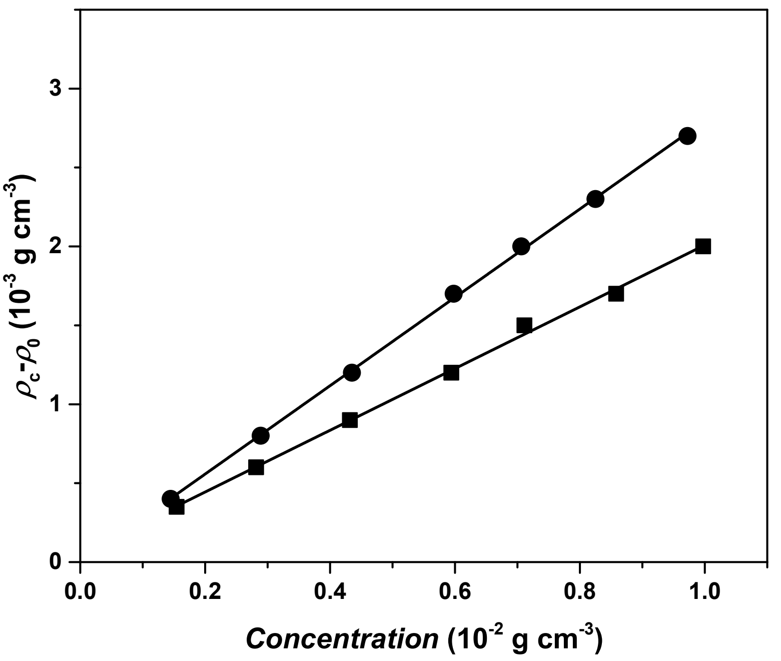

Figure S8. Determination of the apparent partial specific volume, $\boldsymbol{v}$, of objects in solution via densimetry with $\boldsymbol{\rho_c}$ being the density of solutions at specific materials concentration, and $\boldsymbol{\rho_0}$ being the density of the solvent water. The slope of the curves $(\mathbf{1}-\boldsymbol{\rho_0 v})$ was used to calculate the apparent partial specific volume, $\boldsymbol{v}$. Symbols: circles: PVP-*b*-P(OEGMA) in water; squares: PVP-*b*-P(OEGMA) crosslinked via TA in water. The TA-containing system appears more dense in accordance with a lower partial volume, $\boldsymbol{v}$.

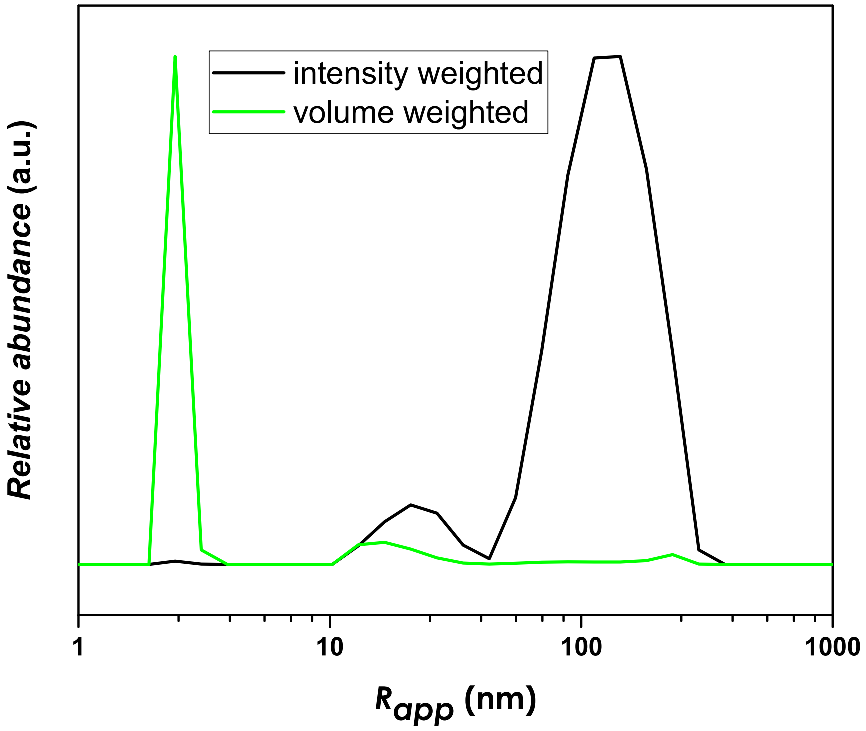

Figure S9. Intensity- and volume-weighted size distributions of PVP-*b*-P(OEGMA) at a concentration of 0.1 wt.% measured via DLS at 25 °C after the crosslinking procedure via TA.

Supporting Information References

1. D. Quemener, T. P. Davis, C. Barner-Kowollik, M. H. Stenzel, *Chem. Commun.* **2006**, 5051

2. J. Willersinn, A. Bogomolova, M. Brunet Cabré, B. V. K. J. Schmidt, *Polym. Chem.* **2017**, *8*, 1244.
